# Airway Problems in Neonates—A Review of the Current Investigation and Management Strategies

**DOI:** 10.3389/fped.2017.00060

**Published:** 2017-03-30

**Authors:** Quen Mok

**Affiliations:** ^1^Pediatric and Neonatal Intensive Care Units, Critical Care Division, Great Ormond Street Hospital for Children, London, UK

**Keywords:** airway, tracheal stenosis, tracheobronchomalacia, bronchography, bronchoscopy, tracheoplasty, internal airway stent

## Abstract

Airway problems in the neonatal population are often life threatening and raise challenging issues in diagnosis and management. The airway problems can result from congenital or acquired lesions and can be broadly classified into those causing obstruction or those due to an abnormal “communication” in the airway. Many different investigations are now available to identify the diagnosis and quantify the severity of the problem, and these tests can be simple or invasive. Bronchography and bronchoscopy are essential to determine the extent and severity of the airway problem and to plan treatment strategy. Further imaging techniques help to delineate other commonly associated abnormalities. Echocardiography is also important to confirm any associated cardiac abnormality. In this review, the merits and disadvantages of the various investigations now available to the clinician will be discussed. The current therapeutic strategies are discussed, and the review will focus on the most challenging conditions that cause the biggest management conundrums, specifically laryngotracheal cleft, congenital tracheal stenosis, and tracheobronchomalacia. Management of acquired stenosis secondary to airway injury from endotracheal intubation will also be discussed as this is a common problem. Slide tracheoplasty is the preferred surgical option for long-segment tracheal stenosis, and results have improved significantly. Stents are occasionally required for residual or recurrent stenosis following surgical repair. There is sufficient evidence that a multidisciplinary team approach for managing complex airway issues provides the best results for the patient. There is ongoing progress in the field with newer diagnostic tools as well as development of innovative management techniques, such as biodegradable stents and stem cell-based tracheal transplants, leading to a much better prognosis for these children in the future.

## Introduction

Development of the airway in the fetus starts from as early as the fourth to fifth week postconception and the structures arise from the branchial arches. There is also an interaction of the blood vessels and the airways during early fetal development of the lungs, with the branching airways forming a template for vasculogenesis (1), hence patients with airway problems frequently have associated lung and cardiovascular abnormalities.

In addition, many airway problems are associated with congenital syndromes and anatomic variants that occur during this embryonic development. Many of these airway problems are now being diagnosed earlier and managed either surgically or in intensive care; hence, the need for neonatologists to be updated about the topic. It is also important for most doctors to be aware of the latest management, as these patients are now surviving into adolescence and early adulthood, thus needing transition of care from pediatricians to pulmonologists and introducing a whole new spectrum of conditions for respiratory physicians to deal with. The purpose of the review is to provide an update on the current investigations available to diagnose the condition, as well as to discuss the various treatment strategies presently available for management of the patient.

## Causes of Airway Problems

Airway problems can be broadly classified into congenital or acquired lesions and further divided into conditions causing obstruction of the airway and those due to an abnormal communication between the airway and esophagus. Congenital obstructive lesions can be from a simple intrinsic lesion in the airway (e.g., hemangiomata, cysts, and webs) or from abnormal development of the airway (e.g., congenital tracheal stenosis). Extrinsic compression can also occur from lesions external to the airway (e.g., vascular rings or slings, lymphangiomatous lesions, mediastinal tumors).

Congenital airway problems due to an abnormal communication (laryngotracheal clefts and tracheoesophageal fistulae) are frequently diagnosed at or soon after birth, because of the more obvious associated abnormalities (e.g., esophageal atresia, facial, and skeletal anomalies) or because of problems during feeding. However, the diagnosis of minor communications (e.g., H-type tracheoesophageal fistula or shorter laryngotracheal clefts) can be delayed, resulting in recurrent aspiration episodes before the diagnosis is eventually made.

Inflammation in the airway (either from infection or injury from endotracheal intubation or recurrent aspiration) can also lead to mucosal swelling causing breathing difficulties due to further reduction in the already small airway lumen. In the longer term, this can cause scarring and narrowing of the airway.

With the advancement of neonatal and pediatric intensive care practices, some of the airway problems we now see are acquired lesions (stenosis or malacia) following instrumentation of the airway and use of positive pressure ventilation or following newer therapies introduced for management of congenital problems. Major tracheobronchial abnormalities had been identified in preterm neonates with bronchopulmonary dysplasia even during the 1980s, with about half of them requiring surgical intervention (2–4). Higher averaged mean airway pressures during the first week after birth and lower gestational age were found to be clinical features associated with tracheomalacia (5). High pressure exerted on a developing airway or chronic inflammation, for example from intubation and mechanical ventilation, may cause weakness of the cartilaginous portion leading to tracheomalacia and acquired tracheomegaly. Recent trials where a balloon occlusion device is introduced into the fetal trachea, in an attempt to increase lung growth in patients diagnosed antenatally with congenital diaphragmatic hernia, have led to focal tracheomegaly from the presence of the device in the developing airway. This results in a large and floppy trachea, which has led to subsequent respiratory difficulties (6, 7).

## Physiology

During normal inspiration there is positive pressure in the airway and negative pressure in the pleural space. During expiration, intra-pleural pressure increases and airway pressure drops as air flow begins, resulting in collapse and possible closure of the tracheal cartilage of a floppy airway (8). This problem is exaggerated during forced expiration or if the patient attempts to increase their expiratory effort, as this will decrease the intra-tracheal pressure more, leading to further collapse and compression of the airway.

Hence patients with obstruction in the upper extra-thoracic airway present with inspiratory stridor, while obstruction in the intra-thoracic part of the airway causes biphasic or an expiratory noise or prolonged expiration on auscultation. In addition, the child may not present until a few months of age, when the obstruction is unmasked by their increased activity and the need for larger tidal volumes as the child grows. Occasionally, the airway problem only comes to light following a respiratory tract infection resulting in increased respiratory effort, or difficulty in intubation with an appropriate size endotracheal tube, or inability to extubate following general anesthesia for a planned surgical procedure. Late presentation at a few months of age does not exclude a congenital lesion.

## Diagnosis

To ensure that a diagnosis is made earlier it helps to have a high index of suspicion. Consider the possibility of an airway problem and investigate if the following symptoms and signs are present:

- recurrent stridor or wheeze
- chronic cough
- recurrent cyanotic episodes
- life threatening events
- feeding difficulties with failure to thrive
- repeated failed extubation attempts
- associated congenital anomalies especially cardiac defects
- signs of dysmorphism
- recurrent aspiration pneumonia
- persistent atelectasis or lobar hyperinflation on chest Xray.

The neonate, who presents with repeated respiratory symptoms, feeding difficulties and recurrent cyanotic episodes, is more likely to have a communication between the airway and esophagus causing recurrent aspiration pneumonia. A history of previous requirement for intubation and mechanical ventilation may provide a clue toward a diagnosis of acquired stenosis, while associated cardiac defect and signs of dysmorphism may suggest a congenital lesion. Repeated failed extubation attempts point toward a diagnosis of airway obstruction due to stenosis and/or malacia. Investigations should therefore be targeted to these possibilities. Patients presenting with cyanotic episodes and life-threatening events should be referred and investigated urgently.

Symptomatic major airway problems are not an uncommon manifestation in preterm babies with chronic lung disease. There has been a recent increase in reports in the literature with earlier recognition due to the ability to investigate the problem (9, 10); as well as an increased occurrence following the use of tracheal occlusive devices in fetuses with congenital diaphragmatic hernia (6, 7).

## Investigations

Simple non-invasive tests are helpful and often provide some clues to the diagnosis, although more invasive tests may often be required to determine the extent and severity of airway involvement. Below are the investigations available to clinicians that can be used in various combinations.

### Pulmonary Mechanics

**Figure 1:**
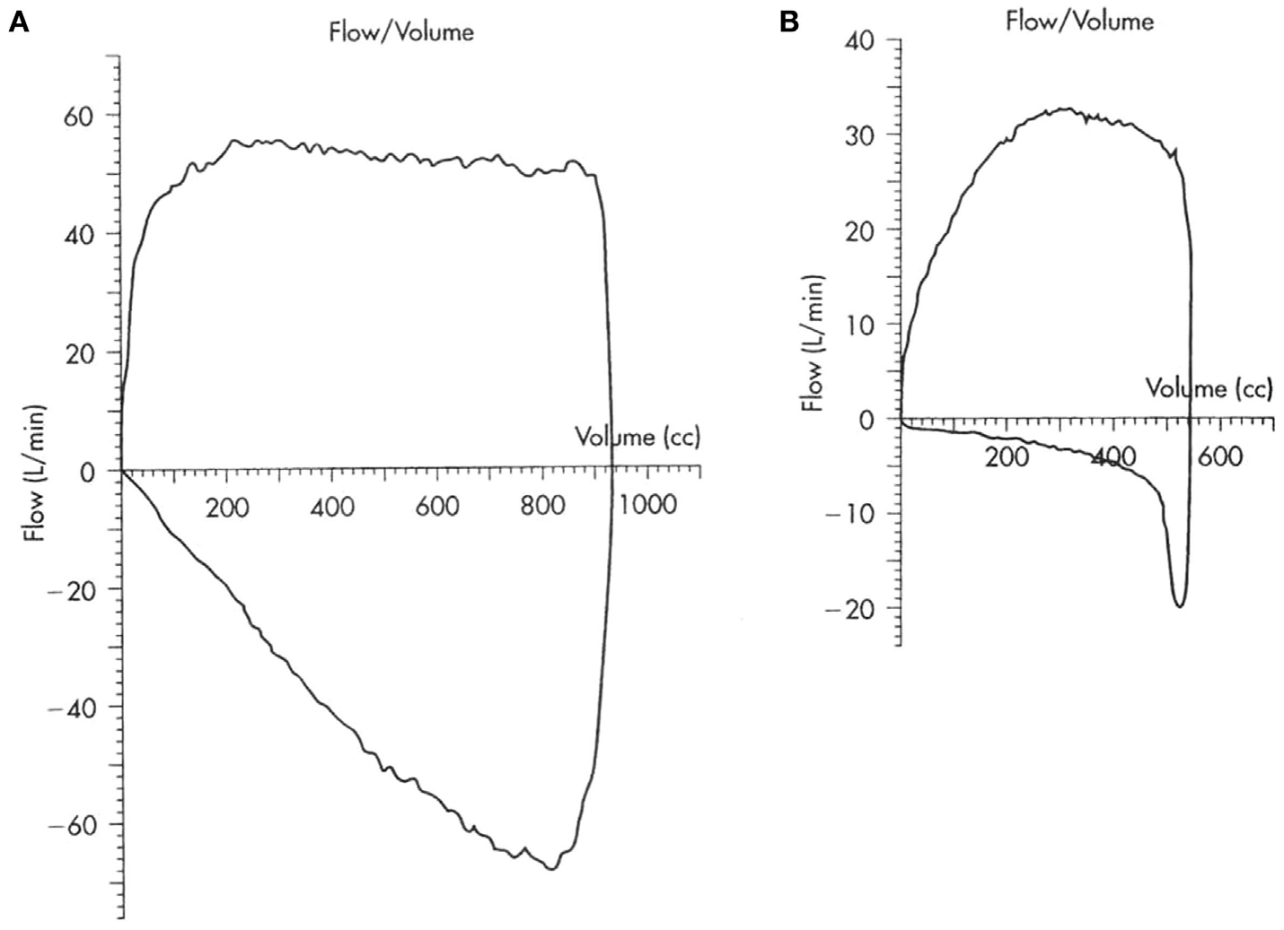
**Flow volume loops in (A) normal and (B) obstructed airway**.

Infant lung function tests have been described although the tests are time consuming to perform and are only available to clinicians in tertiary centers. Otherwise, we would need to wait for the child to be old enough to perform lung function tests, when a low peak expiratory flow rate may be detected to suggest an obstructed airway. However, for the younger child on a ventilator, the diagnosis can be made from the simple pulmonary mechanics and loops that are currently available options in many of the newer ventilators. Figure 1 shows the scalloping of the expiratory limb on flow volume loops typically seen in patients with obstruction in the airway. There would also be evidence of breath stacking in flow pressure waves with the inspiratory phase occurring before completion of the expiratory phase. However, the presence of an endotracheal tube or application of positive end-expiratory pressure may mask an underlying airway problem by stenting open the floppy airway.

### Echocardiogram

An echocardiogram is essential to exclude associated congenital heart disease in patients suspected to have a major congenital airway problem, or in those with dysmorphic features suggestive of various syndromes, as they often have associated cardiac defects. A detailed study should be performed in patients with congenital tracheal stenosis, specifically to look for a vascular sling or ring formed by abnormal configuration of the great vessels causing compression of the airway. Up to a third of the patients with laryngotracheal clefts have associated congenital cardiac abnormality, with a higher incidence with increasing severity of cleft (11, 12).

### Computed Tomography with Angiogram (CTA)

**Figure 2:**
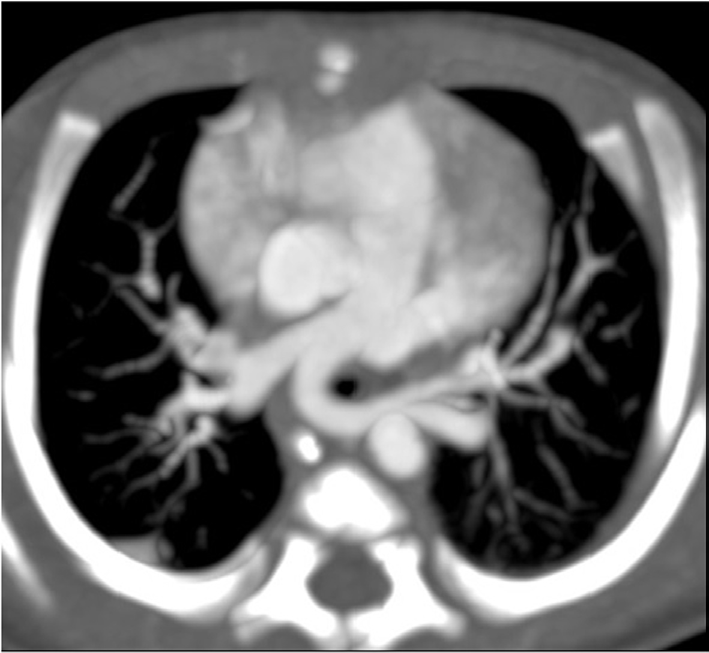
**CT angiogram showing left pulmonary artery sling**.

**Figure 3:**
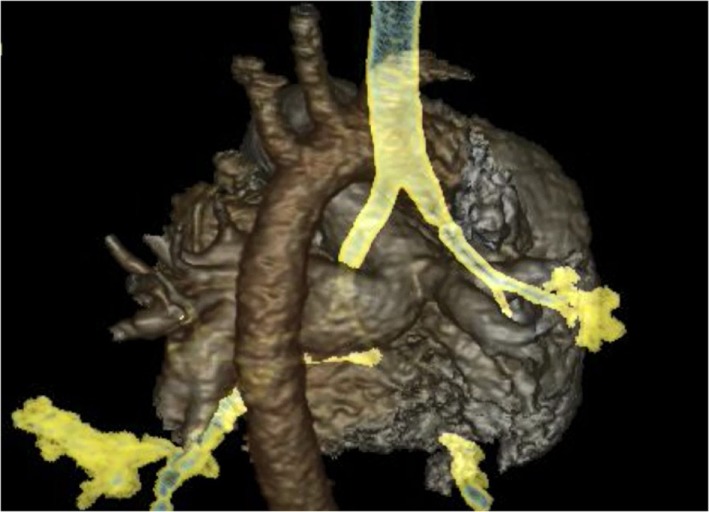
**CT angiogram 3D reconstruction of left pulmonary artery sling**.

High resolution CT scans of the chest with angiogram, especially with 3D reconstructions of the axial scans and virtual bronchography, can be helpful in delineating the airway and vascular anatomy and providing useful data to help plan surgical management. However, one has to consider the radiation dose exposure, which may be an important limiting factor in neonates. Figures 2 and 3 show an axial CTA scan and 3D reconstruction showing compression of the trachea within the sling caused by the anomalous left pulmonary artery arising from the right pulmonary artery. There is often associated abnormality of the tracheobronchial tree and the lung (13, 14), and understanding the airway morphology is important in planning the surgical procedure to be performed in the individual patient.

### Magnetic Resonance Imaging (MRI)

Magnetic resonance imaging is a useful modality for investigation of the pediatric airway and probably is the most accurate modality for defining extrinsic airway abnormalities (15). The radiation exposure in MRI is small compared to CT scanning, and if MRI is available, this would be the preferred modality of investigation in neonates. Currently, cine MRIs can be used to provide real-time dynamic imaging techniques and may therefore be useful in the diagnosis of tracheomalacia (16).

### Tracheobronchography

**Figure 4:**
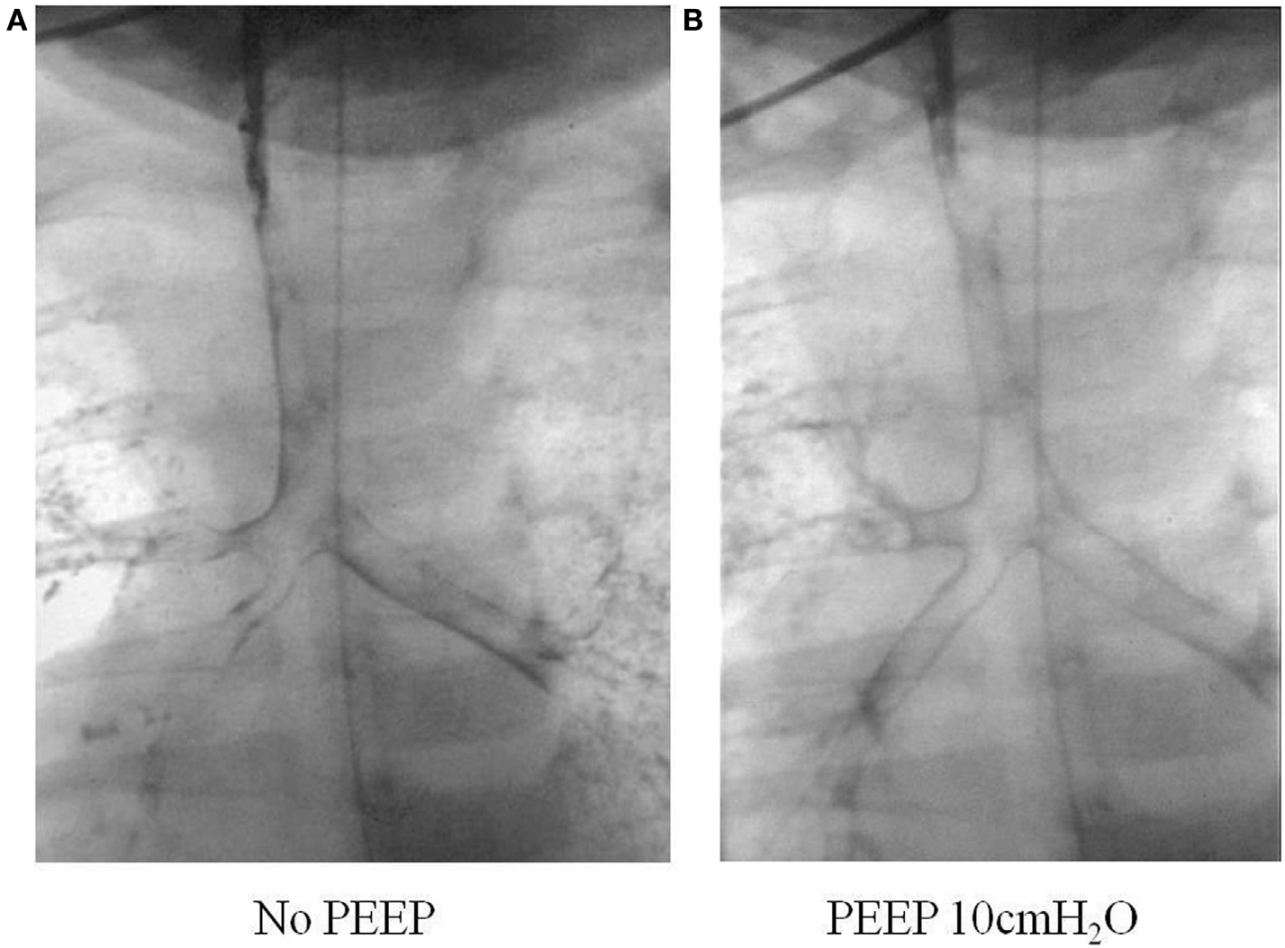
**Tracheobronchogram without (A) and with (B) positive pressure**.

This can be done safely with a small volume of non-ionic water soluble contrast injected into the airway during fluoroscopic screening. One milliliter of Omnipague is usually used *via* a feeding tube inserted into the airway through a laryngeal mask or endotracheal tube. Patients may experience transient and minor desaturations during the procedure, but few other complications occur (17). It is important that the patient is spontaneously breathing as any positive pressure ventilation during the study may mask airway collapse due to malacia. The outline of the respiratory tract is seen, and a manometer can also be connected to the circuit so that the opening pressure can be determined when collapse of the airway has been detected during screening. Figure 4 shows the difference in caliber of the airway with variation in the end-expiratory pressure during a bronchogram study.

We compared the accuracy of diagnosis of airway problems between tracheobronchography and CT scans, which are more widely available, to see if we could obtain as much or similar information from the investigations. We found that bronchography provided superior dynamic information as CT underestimated the presence of bronchomalacia, as well as the severity and extent of tracheal and bronchomalacia (18). A lower radiation dose was also seen with bronchography, although there might be a bigger overlap now with the newer and faster CT scanners.

### Bronchoscopy

**Figure 5:**
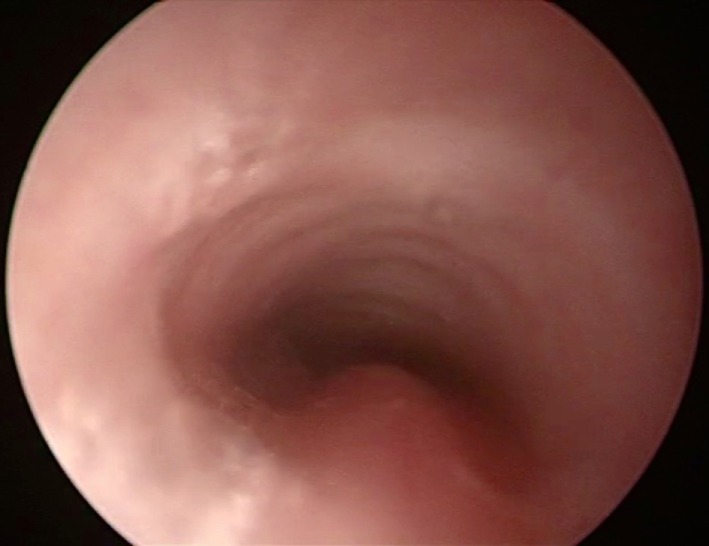
**Bronchoscopy picture of tracheomalacia**.

**Figure 6:**
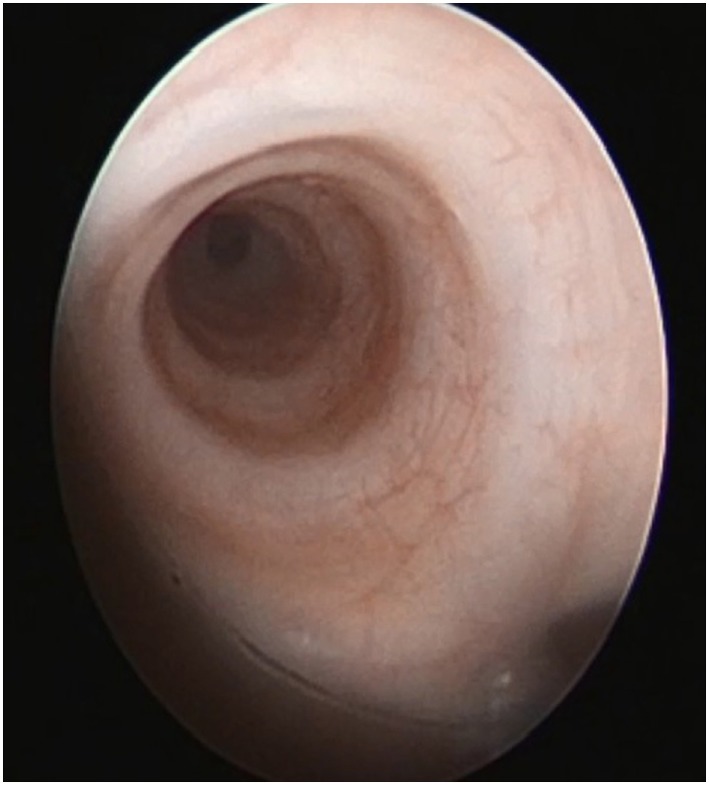
**Bronchoscopy picture of complete tracheal rings in congenital tracheal stenosis**.

**Figure 7:**
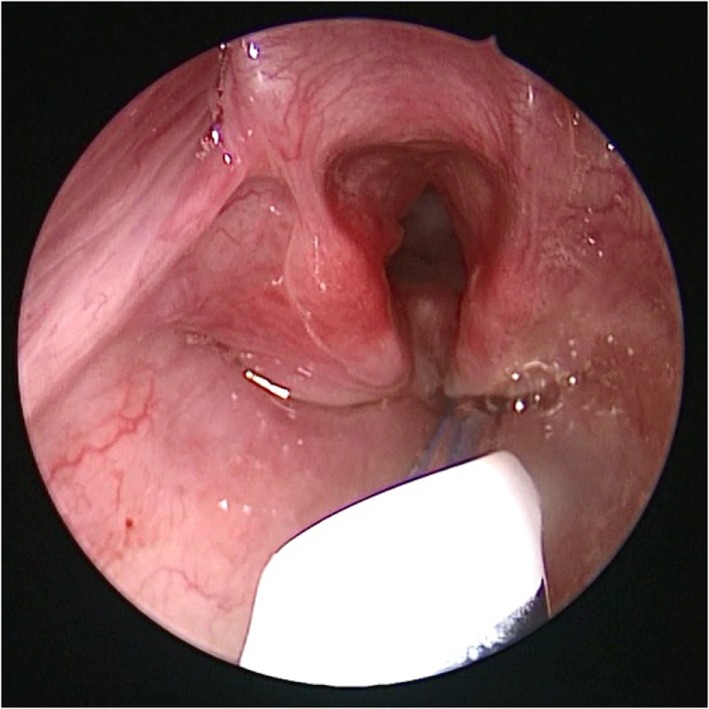
**Bronchoscopy picture of laryngotracheal esophageal cleft, after removal of the endotracheal tube**.

Bronchoscopy may pick up intrinsic airway lesions as well as collapse of the airway during expiration in patients with tracheomalacia (Figure 5). Complete tracheal rings can be seen in congenital tracheal stenosis (Figure 6). This can be done using flexible bronchoscopes at the bedside or rigid ventilating bronchoscopes in theater. A cleft in the larynx may be visualized on direct laryngoscopy during intubation, although this may not be seen when using a flexible scope inserted down the endotracheal tube. More severe degrees of laryngotracheoesophageal clefts may require rigid bronchoscopy to determine the extent of the cleft (Figure 7). For the neonate with small airways, lung disease and very little respiratory reserve, it is unlikely that a bedside study with a flexible scope is possible and rigid bronchoscopy with general anesthetic is likely to be needed in theater.

In this group of preterm patients with chronic lung disease and suspected tracheobronchomalacia causing ventilator dependence, our preferred investigation is bronchography. Not only is the diagnosis made from the investigation but determination of the opening pressure during the bronchogram also helps in management decisions for the infant. We have shown that even a small amount of positive pressure used during the procedure can mask the tracheobronchomalacia and could lead to false negative results (19). The airway can look very normal on bronchoscopy, when positive pressure is applied to a floppy malacic trachea. We stress the importance of making a diagnosis with a dynamic study done during spontaneous breathing effort and without positive pressure ventilation.

### Isotope Ventilation Scan

**Figure 8:**
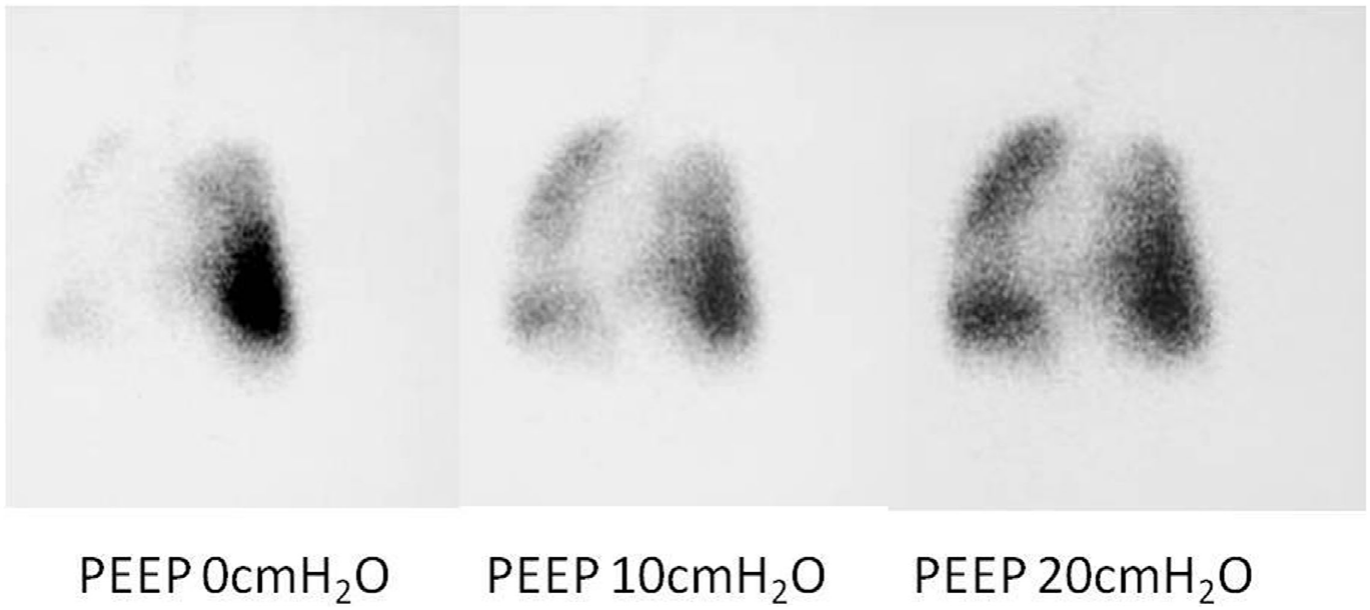
**Ventilation perfusion scan results showing absence of lobar ventilation in the right lung, improving with increasing positive end-expiratory pressure (PEEP)**.

While a bronchogram provides an anatomical picture of the airway, distal bronchomalacia may not be detected on a bronchogram. An isotope ventilation scan can be a valuable tool in the diagnosis and management of patients with distal bronchomalacia with persistent atelectasis seen on chest Xray. Unilateral or focal areas of absent ventilation can be shown to improve with increasing levels of positive pressure (Figure 8), hence providing physiological images of the lungs at known levels of positive pressure (20). This could help to determine the ventilator pressures required to ensure all areas of the lung are adequately ventilated.

## Management

Subsequent management needs to be planned following careful precise assessment of the airway and may need to be highly individualized (21). Not all patients will need surgery following the diagnosis, and some patients can be managed with a “watch and wait” approach, as long as they are putting on weight and do not have life-threatening symptoms. The need for, and type of, intervention depends on the extent and severity of airway involvement, which would often determine how symptomatic the child is.

### Tracheal Stenosis

#### Surgery

Acquired tracheal stenosis caused by the endotracheal tube often involves a short segment of the subglottis. Milder degrees can be treated early with an anterior cricoid split, which has been successful in facilitating extubation and avoiding the need for a tracheostomy (22). However, for higher grades of stenosis, surgical laryngotracheal reconstruction performed as a single stage using cartilage graft is the preferred treatment option (23, 24). This can be done with minimal morbidity and a high success rate. Postoperative management can be successfully carried out without physical or pharmacological restraints, resulting in less adverse events and a shorter length of stay in intensive care and in hospital (25).

Congenital tracheal stenosis can be localized or affect variable lengths of the airway, occasionally involving the carina and extending into the bronchus. An unusual arborization pattern of the trachea and bronchi has been described in patients with congenital tracheal stenosis, with tracheal right upper lobe bronchus and trifurcation pattern being the most commonly seen morphology (13). Surgery is indicated if the patient is symptomatic, and this is often related to the degree of airway narrowing. Shorter segments of stenosis can be treated with tracheal resection and direct anastomosis, although tracheal reconstruction would be needed for extensive stenosis. Management can be challenging if a long segment of the trachea is involved. The earlier results from surgery were poor with a high mortality (26, 27), resulting in different surgical techniques being tried, including patch tracheoplasty and external Hagl stents. Slide tracheoplasty is the current preferred surgical option for long-segment tracheal stenosis, which allows for surgical widening of the airway using the patient’s own airway (28–30). The procedure frequently leads to a shortening of the length of the trachea, but the reconstructed trachea has been shown to grow, especially in the first 6 months after surgery (31). If there is an associated cardiac abnormality causing compression of the airway (e.g., pulmonary artery sling or double aortic arch causing a vascular ring), this is often repaired at the same time and cardiopulmonary bypass is required.

Following repair, the airway is assessed using bronchoscopy a week after surgery, and balloon dilatation may be required for stenotic areas. We try to aim for the child to be extubated as soon as possible to reduce the complications of prolonged instrumentation of the airway, and it has been shown that there are fewer complications if the child is not kept sedated and muscle relaxed on mechanical ventilation (25). Patients are monitored regularly in the early postoperative period, and frequent airway assessments with bronchography and bronchoscopy may be required in the first few weeks for early detection and management of airway obstruction from granulation tissue formation (28).

#### Stents

Airway stents may be needed following surgery if there is recurrent stenosis despite balloon dilatation. These have been used for treatment of obstruction since the 1990s, and can be inserted easily and safely and left in place for prolonged periods to relieve major airway obstruction (32), and have allowed the patient to be managed without mechanical ventilation. Stents have evolved from fixed shapes and sizes to balloon expandable stents originally used for angioplasty, and the stent materials used have changed from metallic to silastic to biodegradable material (33–35). Granulation tissue formation occurs and may require bronchoscopic manipulation with balloon dilatation and early stent removal. Displacement of the stent and erosion into surrounding tissue are rare but life threatening complications. Biodegradable polydioxanone stents are currently available and are custom made for the patient. They have the advantage of avoiding permanent stenting or the need for removal, allowing for subsequent growth of the airway (35).

Patients who require stents have more complications, with the need for more frequent intervention, delay extubation, and are associated with more infections. Airway surgery and the subsequent use of stents are a significant risk factor for microbial colonization of the airway, and airway stents specifically increase colonization of the distal airway (36).

### Tracheobronchomalacia

Tracheomalacia results in collapse of the floppy cartilaginous portion of the airway especially during increased respiratory effort. Focal malacia can be stented in an attempt to get the patient off the need for positive pressure support. However, if tracheomalacia is severe or extensive, the recommendation is for tracheostomy to facilitate maintaining patency of the airway with continuous positive airway pressure (CPAP). The malacia in preterms neonates tends to be extensive and involve most of the tracheobronchial tree. Although the airway improves with time as the child grows, this could take months, and these patients are best managed with tracheostomy and CPAP as this allows for discharge from intensive care and improves neurodevelopmental outcome.

Aortopexy may be successful in relieving symptoms in those children who have persistent respiratory problems due to tracheomalacia following surgical repair of tracheal stenosis or tracheoesophageal fistula. Pulling the aorta anteriorly and away from the tracheal wall may be effective in reducing symptoms caused by tracheal compression. It is a safe and effective procedure (37, 38). There is, however, a lack of general consensus for the indications for aortopexy, although acute life threatening events is often an absolute indication (39). The mortality is low and the efficacy is high with resolution of the symptoms in the majority of cases. Major comorbidity is associated with an adverse outcome with a longer length of ventilation and ICU stay, higher mortality and the need for further procedures (redo surgery, tracheostomy, and internal stents) in these patients (40).

### Laryngotracheoesophageal Clefts

This is a rare congenital abnormality in the posterior laryngotracheal wall, leading to a communication with the esophagus. These have been classified into type I–IV by Benjamin and Inglis depending on the length of the cleft and the extension into the glottis, cricoid cartilage, or the cervical or thoracic section of the trachea (41). The minor type I or II clefts can be easily repaired endoscopically or through the neck, with good results although patients frequently have swallowing difficulties. However, there is often associated serious comorbidity with the more severe clefts leading to early mortality. Those patients with extensive clefts where surgical repair is attempted frequently require repeated operations, hence good liaison and team working between cardiothoracic, otorhinolaryngology, and intensive care teams are important (12, 42).

## Prognosis

**Figure 9:**
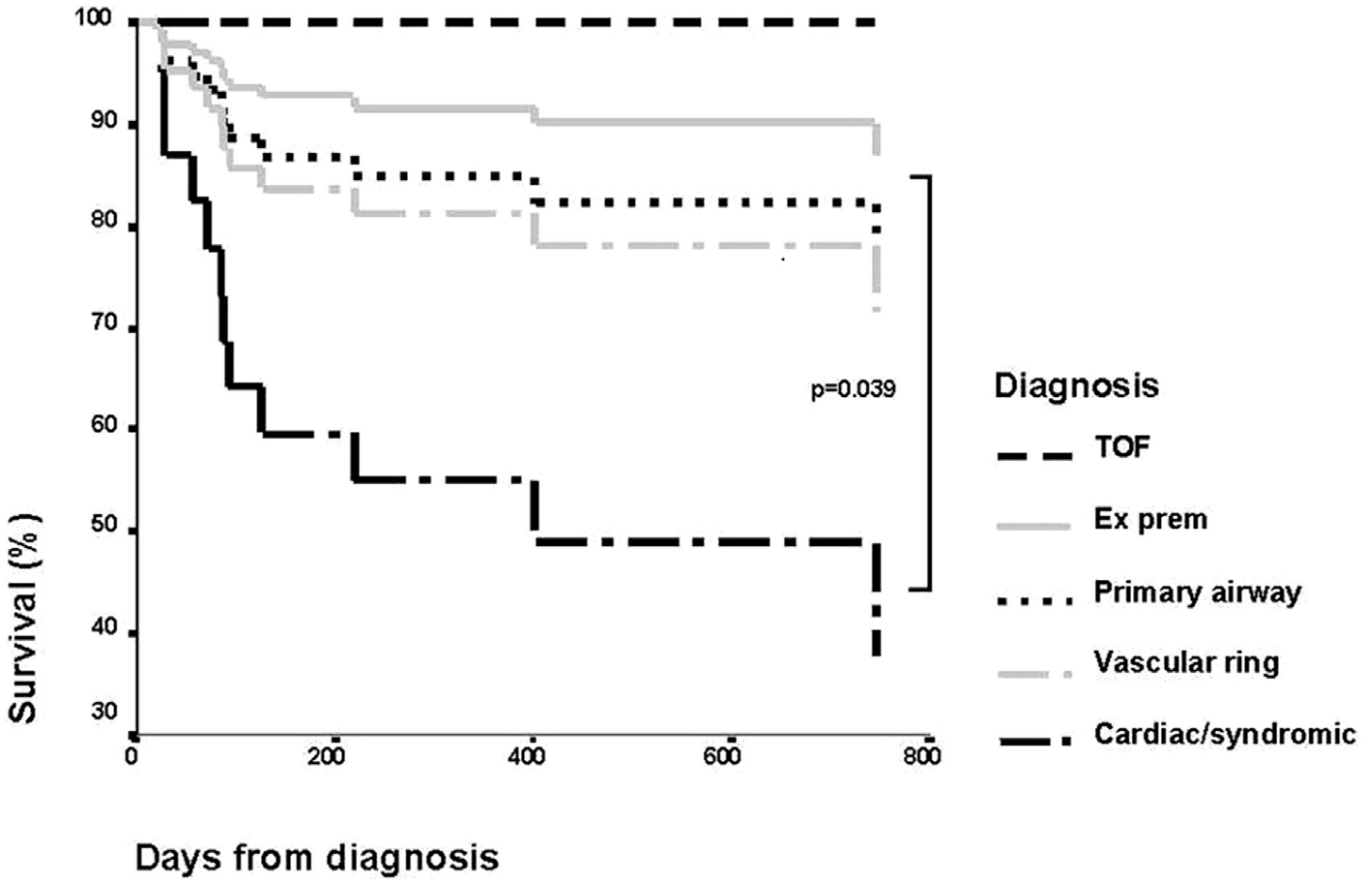
**Kaplan–Meier curve showing survival of patients by diagnostic group**. Reproduced with permission from Springer and Intensive Care Medicine.

The outcome of children with tracheal stenosis and tracheomalacia has improved significantly in the last few years. The mortality was previously high with significant morbidity. It was initially reported that the need for ventilation for longer than 14 days and the bronchogram findings of moderate to severe malacia of the trachea and bronchi were predictive of a fatal outcome (43). Review of our patient population, however, showed that length of ventilation or severity of tracheomalacia was not predictive. Figure 9 shows the Kaplan–Meier curve of survival in different diagnostic groups. We found that associated comorbidity was more predictive of mortality, and patients with cardiac problems or who were syndromic had a much poorer outcome than patients who had tracheomalacia associated with trachea-esophageal fistula, as an isolated primary airway problem or from prematurity (44).

**Figure 10:**
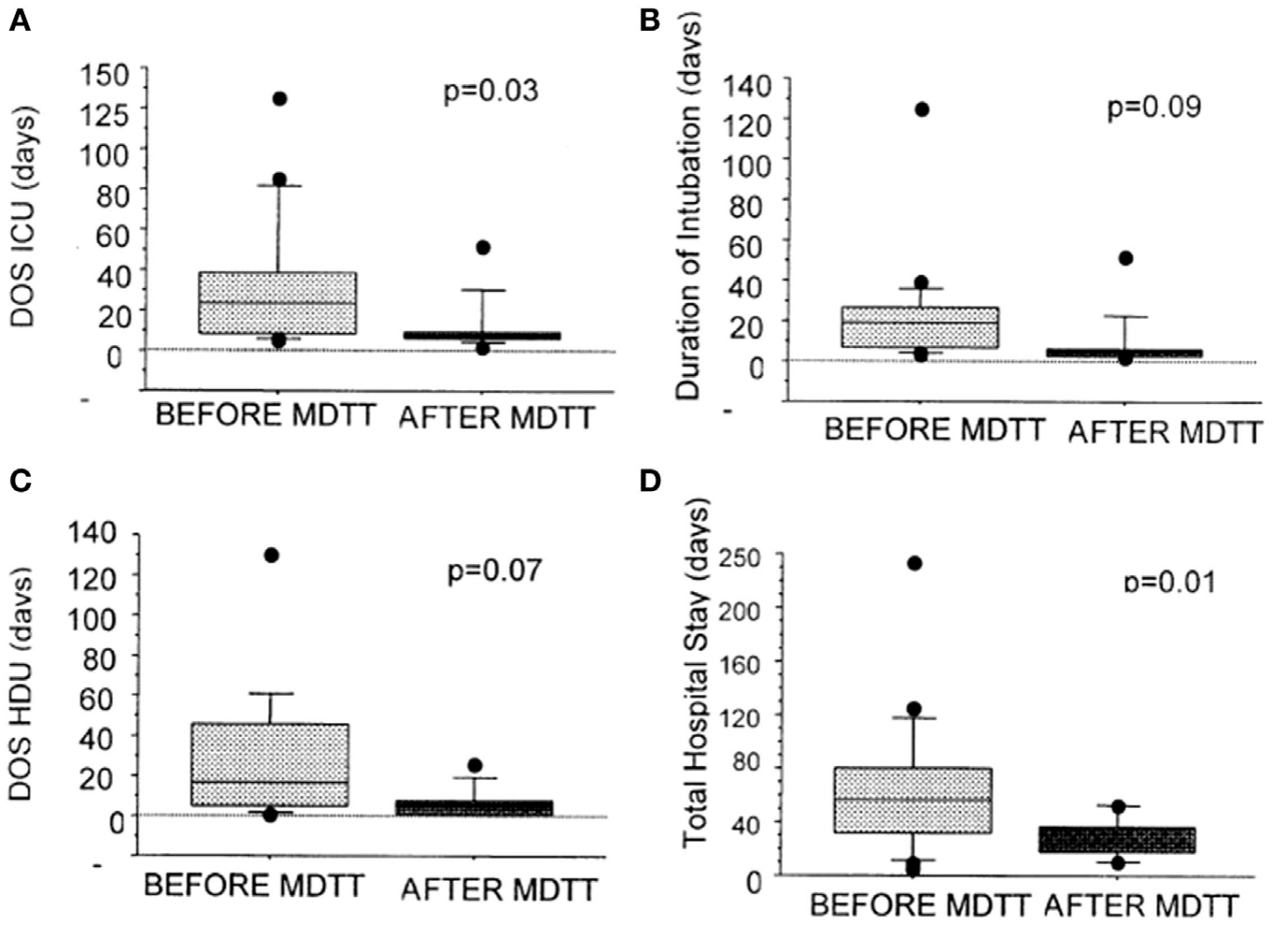
**Graphs showing the effect of the introduction of multidisciplinary trachea team working on duration of ventilation, length of ICU, and hospital stay**. Reproduced with permission from Elsevier and The Journal of Thoracic and Cardiovascular Surgery.

Further improvements have occurred with advances in surgical and interventional radiology techniques, intensive care management, and multidisciplinary team working in centralized units (21, 42, 45). Figure 10 shows a reduction in length of ventilation, in addition to a significant reduction in the length of stay in ICU and in hospital. The costs for management per complex airway patient have therefore come down significantly by £51,114 per patient (46). The long-term survival now exceeds 88%, and many patients have a normal quality of life (21).

## Future Directions

Stem cell-based tissue-engineered tracheal replacement is an exciting therapeutic option for the future. A decellularized cadaveric tracheal scaffold is seeded with bone marrow mesenchymal stem cells harvested from the recipient. This has been done in 2010 in a child who had tracheal stenosis and failure of surgical repair with erosion of the stent. There is evidence of revascularisation of the graft within a week after surgery and restoration of the respiratory epithelium 1 year later. After 18 months, the graft had biomechanical strength, and the patient has not needed any medical intervention since. His CT scan and ventilation perfusion scan are reported as normal, and he has grown 11 cm in height since the procedure. He has been followed up 6 years posttransplant and now leads a completely normal life (47, 48).

## Conclusion

In patients with congenital tracheal stenosis, careful assessment of the airway is of paramount importance to plan further management strategies. We feel that bronchography and flexible bronchoscopy are essential tools for diagnosis, intervention, and follow-up, although echocardiography, computed tomography, and MRI may be important for the evaluation of associated cardiovascular abnormalities. Our current management strategy involves slide tracheoplasty in combination with balloon dilatation and occasionally stenting in cases of recurrent stenosis. Multidisciplinary team working within centralized units is crucial and has reduced the morbidity and cost of managing these patients. Survival rates have risen dramatically, and innovative management strategies will lead to a much brighter outlook for these patients.

## Author Contributions

QM is the sole author who researched the topic and wrote the entire paper. Some of the radiographs and bronchoscopy pictures have been provided by the other members of the trachea team.

## Conflict of Interest Statement

The author is a consultant intensivist working in Great Ormond Street Hospital in the National Health Service. She is a member of the multidisciplinary trachea team of the hospital, although has no additional time or remuneration for being involved in this team.
